# High individual consistency in fear of humans throughout the adult lifespan of rural and urban burrowing owls

**DOI:** 10.1038/srep03524

**Published:** 2013-12-17

**Authors:** Martina Carrete, José L. Tella

**Affiliations:** 1 Department of Physical, Chemical and Natural Systems, University Pablo de Olavide, Ctra. Utrera km 1, 41013 Sevilla, Spain; 2 Department of Conservation Biology, Estación Biológica de Doñana (CSIC), Avda. A. Vespucio s/n, 41092 Sevilla, Spain

## Abstract

Human-induced rapid environmental changes challenge individuals by creating evolutionarily novel scenarios, where species encounter novel enemies, the new species sometimes being humans themselves. However, little is known about how individuals react to human presence, specifically whether they are able to habituate to human presence, as frequently assumed, or are selected based on their fear of humans. We tested whether fear of humans (measured as flight initiation distance in a diurnal owl) is reduced through habituation to human presence (plasticity) or whether it remains unchanged throughout the individuals' life. Results show an unusually high level of individual consistency in fear of humans throughout the adult lifespan of both rural (r = 0.96) and urban (r = 0.90) birds, lending no support to habituation. Further research should assess the role of inter-individual variability in fear of humans in shaping the distribution of individuals and species in an increasingly humanized world.

Two-thirds of the world's terrestrial area is now dedicated directly to support human populations, either through agriculture, fisheries, urbanization, or infrastructure^1^, imposing rapid human-induced environmental changes on wildlife. From the local to the global scale, human actions have influenced – and in many cases, disrupted – the structure and functioning of populations, communities, and ecosystems, with several examples of human-induced changes in wild populations^2^,^3^,^4^,^5^,^6^. Traditionally, the most adverse human activities for fauna have been related to natural landscape modification. However, a growing number of studies are now showing that human presence can alter animal activities through behavioural changes. Behaviour appears to be important in explaining variation in species' abilities to cope well with human-induced habitat changes, with maladaptive behaviours leading to species decline and more appropriate behavioural responses facilitating persistence and even range expansion^7^. Different studies have suggested that behavioural flexibility *per se* helps species to cope with rapid human-induced environmental change^8^,^9^,^10^. Recently, however, other research has supported within-species (i.e., inter-individual) variability in certain behaviours that can allow species to cope well with this kind of environmental variation^11^,^12^,^13^.

Rapid human-induced environmental changes place organisms in evolutionarily novel scenarios characterized by more rapid conversions than they have experienced during their evolutionary past^7^. Behavioural responses to these changes include, among others, avoiding or coping with novel enemies, with the new species in some cases being humans themselves. Flight initiation distance (hereafter, FID), the distance between an approaching human and a focal animal at which the latter flees, provides a standardized estimate of fear of humans^14^. For that reason, FIDs have been used to establish buffer zones for minimizing human disturbance^15^, often suggesting that populations with short FIDs have habituated to human presence^16^,^17^,^18^. However, studies on FID have overlooked whether individual FID would change through habituation or, conversely, whether it remains constant throughout the lifespan of an individual. Recently, Carrete & Tella^19^ showed that FID shows a high short-term, within-year individual consistency (repeatability = 0.84–0.92) in a bird species, suggesting that this behaviour could determine how individuals distribute themselves in the habitat depending on their susceptibility to human disturbance. Extending this hypothesis to the invasion of urban environments, the most populated habitats worldwide, a further comparative study concluded that urban invaders are the tame individuals of species showing large inter-individual variability in their fear of humans^12^. Thus, populations with short FIDs can not only arise through habituation (i.e., behavioural plasticity) but also through selection processes, provided that this behaviour remains constant through the lifespan of the individuals and has a heritable component. An important step in understanding the strength and evolutionary consequences of selection acting upon fear of humans is therefore to document how much of this behaviour remains constant across time and contexts (animal ‘personality’), and how much corresponds to individual responsiveness to environmental variation (plasticity)^20^. In this sense, repeatability is a measure commonly used to quantify the constancy of phenotypes, expressing the proportion of phenotypic variation (i.e., the amount of intra-individual variation) in a trait relative to the total phenotypic variation (i.e., the sum of inter-individual and intra-individual variation). However, this analysis does not provide information on the level of individual plasticity within a population^20^. This point, i.e. the possibility that individuals from the same population differ in their degree of behavioural plasticity^20^,^21^, is an important aspect that should be explored as it can affect predictions of evolutionary change in response to selection^22^,^23^.

Here, we investigated long-term individual variation in fear of humans taking advantage of a large data set of individually marked burrowing owls (*Athene cunicularia*) occupying rural and urban habitats in Argentina. The two habitats greatly differ in their degree of human presence, with marked differences in fear of humans among individuals occupying them^12^. This allowed us to evaluate the repeatability of this behaviour in individuals breeding in habitats subject to very different ecological pressures, while exploring their sources of variability. Specifically, we assessed the relative contribution of behavioural stability and behavioural plasticity in fear of humans through the adult lifespan of individuals. Individual variation in both the average level of behaviours and behavioural plasticity has been observed among subpopulations of great tits (*Parus major*), suggesting that individual variations in some behaviours such as exploration may largely be shaped by mechanisms acting within populations^23^. We thus examined possible differences among individuals in their average level of fear and their long-term behavioural plasticity using a reaction norm approach. This approach considers that the phenotype of an individual can be expressed in different environments (or moments) as a line, described by an elevation (the individual's level of behaviour in the average environment; “intercept” in statistical terms) and a trend (the individual's plasticity over an environmental or temporal gradient; “slope” in statistical terms). When there are no significant between-individual variations in slopes (individual × environment interaction or “I × E”), the variance attributable to the individuals (individual variation or “I”) can be used to estimate the repeatability of a trait. However, if individuals also vary in their plastic response to the environment (i.e. slopes), then the among-individual variance for the trait will necessarily change across environmental conditions, complicating the definition and measurement of repeatability^20^,^24^. Our results provide the first evidence for a high level of consistency in fear of humans throughout the individual adult lifespan, suggesting minimal plasticity in this trait.

## Results

We recorded a total of 536 FIDs in 338 banded adult owls over five consecutive breeding seasons. According to their short-term life expectancy (1.3–2.9 yrs), most individuals (n = 202) were tested for FID in only one year, while inter-annual repeated measures were available for 136 birds (84, 42 and 10 individuals tested 2, 3 and 4 consecutive years, respectively; no birds were apparently alive after 4 years). FIDs greatly varied among individuals, ranging from 3.5 to 130 m.

**Figure 1:**
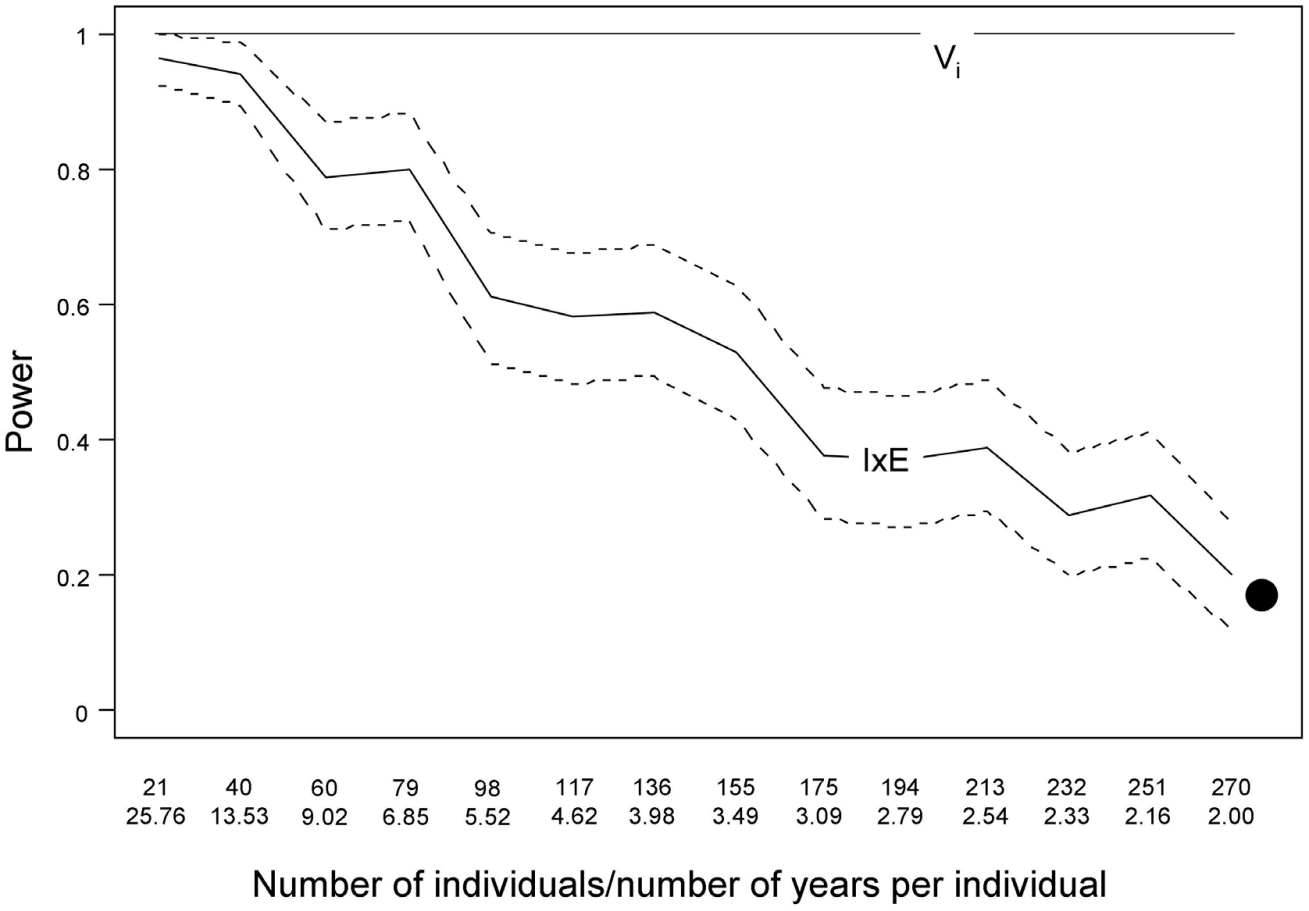
Power of GLMMs to detect individual variation (V_i_) in FID and between-individual variations in slopes (individual × environment interaction or “I × E”) for a total sample size of 540 observations with varying repartition of observations between individuals and number of years of observation per individual. Solid lines represent mean values of the simulations and dashed lines the 95% CI.

Models to analyze the long-term variability in fear of humans that included individuals nested within territories showed slightly lower AIC values than those including only individuals as random terms (ΔAIC_REML_ = 2.68, Table 1), supporting a slight effect of the local environment (territory) on FID. Regarding individual plasticity in fear of humans, we found that the random intercept model was more appropriate than the random intercept and slope model (likelihood ratio test: χ^2^_(df = 2)_ = 2.84, p = 0.2417). Thus, even when adult birds varied in their average level of fear of humans (Vi), their long-term responses were similar (no IxE effect). However, our data structure (338 individuals with ca. 2 measured years per individual) yields an estimated statistical power of 1 to detect V_i_ but just 0.18 to detect IxE (Fig. 1).

**Figure 2:**
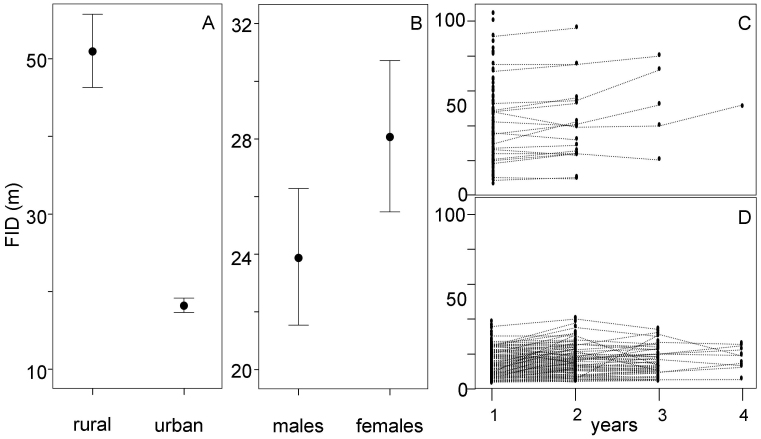
Differences in fear of humans (measured as FID, in m; mean and 95% CI) between individuals occupying rural and urban habitats (A) and between males and females (B). Variation in fear of humans over successive years in rural (C) and urban (D) individuals.

FID was highly repeatable within individuals over successive years (r = 0.85), significantly exceeding the value obtained in simulations (Table 1). This repeatability was slightly higher (r = 0.91) when individuals were nested within territories (Table 1). The best supported model (in terms of AIC_ML_) to explain variability in FID indicated that sex of birds, the habitat where they lived and the successive years birds were tested affected this behaviour (Table 1). Owls breeding in urban habitats showed less fear of humans than those occupying rural landscapes (FID of urban birds: 18 (SD 9.59) m, n = 235; FID of rural birds: 51 (SD 26.99) m, n = 103; F_1,146_ = 273.43, p < 0.0001; Fig. 2A). Moreover, males had lower FID than females (23.91 (SD = _19.42) m, n = 163, and 28.10 (SD = _22.13) m, n = 175, respectively), although differences were not statistically significant (F_1,146_ = 2.58, p = 0.11; Fig. 2B). Interestingly, FID of individuals slightly increased across years (F_1,146_ = 4.41, p = 0.0375; estimate: 0.03, SE = 0.01; Fig. 2C).

The repeatability in fear of humans was even higher when FIDs of urban and rural birds were modelled separately (r = 0.90 and 0.96, respectively, Table 1, Fig. 2C), and the positive trend in FID across years remained significant for rural (F_1,24_ = 9.59, p = 0.0049) but not for urban (F_1,129_ = 2.73, p = 0.1009) birds. The variance among rural territories seems to have had a reduced effect on individual FIDs since models including the individual as random terms were much better supported than models fitting individual nested within territory (ΔAIC_REML_ = 14.66, Table 1). However, this result could also be partially due to the fact that we obtained repeated measures of FID within territories in a lower percentage of rural (27%) than urban (58%) territories.

**Figure 3:**
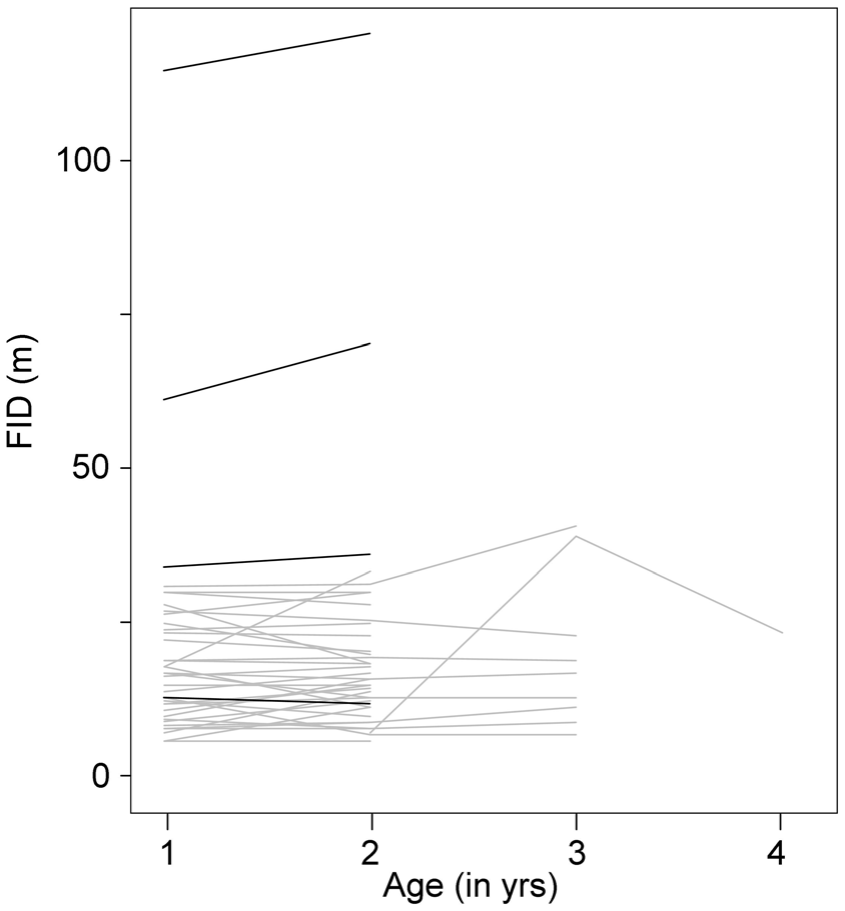
Fear of humans of individuals tested since their first breeding (as one yr–old birds) and through their lifespan. Black and grey lines represent rural and urban individuals, respectively.

The subsample of adults banded as chicks and tested for FIDs since their first reproduction (n = 38) showed significant differences between rural and urban birds (F_1,46_ = 16.67, p = 0.0002) but no sexual (F_1,46_ = 2.59, p = 0.1146) or age (F_1,46_ = 2.14, p = 0.1507) effects. Although the two latter effects might have lost statistical significance due to the smaller sample size, the lack of a significant age effect may also result from a bias in sample size towards urban birds (34 urban and 4 rural birds), whose FID did not change across years. Regardless, it is worth noting that individuals showed no signals of habituation to humans from their first reproduction through their adult lifespan (Fig. 3).

## Discussion

Although behaviour has often been considered highly plastic, there is growing awareness that individuals not only show limited behavioural plasticity, but that individuals from the same population can also differ in their degree of behavioural plasticity^20^,^21^. Therefore, it is a top priority to ascertain whether and to what extent individuals differ in their behavioural plasticity^20^,^25^. Behavioural plasticity has been recently conceptualized and measured in terms of reaction norms, relating individual behaviours to an environmental gradient. In our case, the environmental gradient is represented by an accumulated exposure of individuals to human presence over years (thus matching a temporal gradient). A key emerging result considering the reaction norm approach is that even when owls greatly differ in their average level of fear of humans (individual FIDs ranging from 3.5 to 130 m), both tame and fearful individuals behaved consistently throughout their adult lifespan.

However, to what extent can we attach biological meaning to a reaction norm that is statistically unsupported? A slight reaction norm was obtained when studying short-term variability (i.e., within a breeding season) in the FID of rural burrowing owls^17^, but results are only marginally significant when reanalyzed and applying the most recommended likelihood ratio test^42^ (p = 0.056). Although a reaction norm is not supported across the adult lifetime of individuals, the power to detect an IxE effect is very low and thus we cannot completely discard the existence of some inter-individual variability in plasticity in fear of humans. Simulations suggest that sampling strategies to achieve sufficient statistical power to detect IxE at biologically plausible levels require an increase in the number of monitored individuals or the number of replicates per individuals. Increasing the number of replicates (years) is impractical in our study species, due to its adult lifespan, while increasing the already large number of birds that were monitored (n = 338) is logistically difficult. Regardless, the high repeatability of fear of humans over years (r = 0.85–0.96), compared to other animal behaviours (r ~ 0.4–0.5)^26^,^27^, suggests that the margin left for any (statistically undetected) existing inter-individual variance in plasticity would be very small. However, rural but not urban owls showed a slight increase in FID with years when analysed separately, thus suggesting that these “populations” slightly differ in their degree of plasticity, which may be undetected by the reaction norm approach. Although we have no clear hypothesis to explain the unexpected positive trend in FID across years in rural owls, the important point here is that it runs counter to the frequently claimed habituation process.

Differences in fear of humans among populations have been traditionally explained through habituation^16^,^17^,^28^,^29^,^30^, where animals reduce their FIDs by a learning process in which the stimuli cease to be regarded as dangerous after repeated exposures to it^31^. Hence, animals would allow humans to approach more closely before fleeing in subsequent encounters. Although the role of habituation is commonly accepted in FID studies, alternative mechanisms could produce the same prediction^6^,^17^,^19^,^32^. Here we have found a pattern of shorter FIDs in urban compared to rural owls similar to that expected under habituation. However, the mechanisms producing this analogous pattern seem to be rather different. Contrary to previous studies, these results are based on individually marked birds that were monitored across years, thus allowing us to affirm for the first time that fear of humans remains highly constant throughout the adult lifespan of a bird species. Thus, differences among urban and rural populations of burrowing owls are more likely a consequence of selective pressures (e.g., precluding frightened individuals from colonizing urban areas or favouring their emigration from urbanized areas^12^), lending no support to the idea that individuals living in contact with humans reduce FID through habituation.

It is worth noting that our study focused on the adult lifetime of individuals, and thus we cannot discard parental or early experience effects^33^ influencing the FID of burrowing owls during their few months of life before acquiring a territory and mate for breeding (see Study Species). Although nothing is known for FID, it has been shown that other risk-taking behaviours have a heritable component in vertebrates^34^. While much more research is needed to generalise and fully understand the ecological and evolutionary implications of our results, it is advisable to consider the possibility that variability in fear of humans among individuals of some species would be subject to selection, through genetic and/or non-genetic inheritance^35^,^36^,^37^, and that intra-population variability in this behaviour could be an important aspect of the persistence of species in environments with human presence^12^.

## Methods

### Ethic statements

Capture, banding and FID measures of Burrowing owls were conducted under permits from the Argentinean wildlife agency (22500-4102/09), the Ethic Committee of CSIC (CEBA-EBD-11-28), and the owners of private properties.

### Study species

The burrowing owl (*Athene cunicularia*) is found across American open landscapes, showing diurnal activity and nesting in burrows excavated by the owls or by mammals^38^. Breeding pairs are territorial and highly conspicuous in the daytime, and are easily located usually within 30 m of their nests^17^. The species has declined in many parts of its northern range^39^ but remains abundant in grasslands and even urban habitats of South America^12^. Published adult survival rates^40^ were used to estimate the average adult lifespan of the species (as −1/ln(survival)^41^), ranging between 1.3 and 2.9 years. This figure seems reasonable given the number of years that individually-marked individuals were resighted in this study (see results). In our study area, burrowing owls fledge from nests from late December to the end of January, are associated with parents for at least one month, and acquire territories and mates for breeding in September-October (when they are less than one year old) (authors, unpubl. data).

### Field procedures

**Figure 4:**
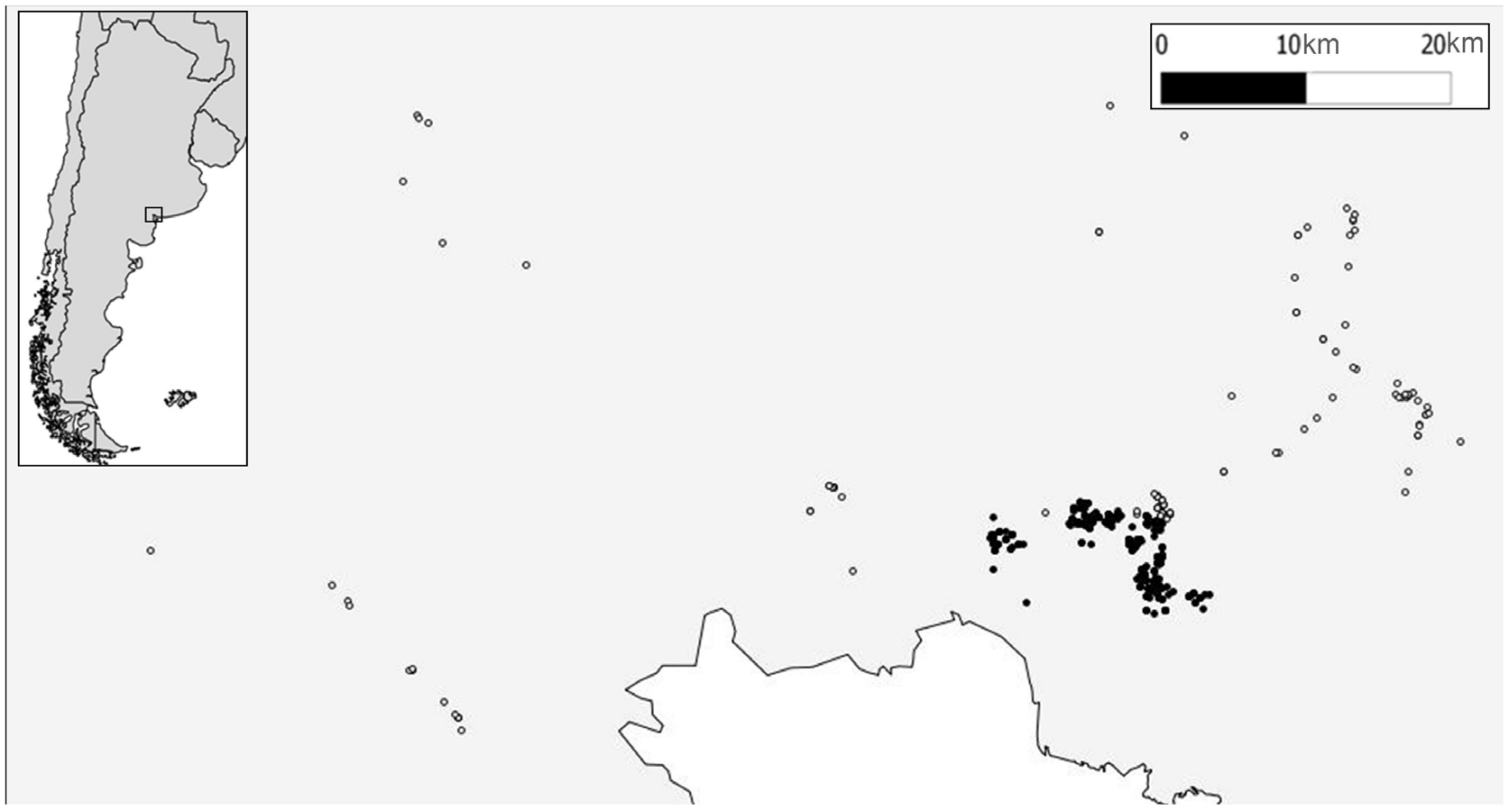
Study area and spatial distribution of urban (black dots) and rural (white dots) territories where adult breeding burrowing owls were tested for FID behaviour. Map generated by MC using Q-GIS 1.8.0 (2008 Free Software Foundation, Inc).

We annually GPS-located up to 480 nests of burrowing owls in a 4,000 km^2^ area comprising rural and urban areas around Bahía Blanca (Argentina), a young city reaching 293,000 inhabitants in recent years^12^. Rural habitats are mostly composed of large extensions of natural grasslands and pastures devoted to wide-ranging livestock and low-intensive cereal crops, with small, interspersed patches of xerophytic forests and scrublands. Human presence is extremely low and mostly restricted to a few paved or unpaved roads (with 0–0.1 pedestrians/h and 0.34–2.4 cars/h)^12^, since nearly all of the area belongs to private properties (with each being several thousands of hectares) which are fenced and thus pedestrians are not allowed to freely walk through the countryside. Therefore, most owls breeding in rural habitats have little or even no close contact with humans (or only with the researchers). This strongly contrasts with urban owls, which excavate their nests in small (usually 0.01–0.1 ha) private and public gardens in urbanized residential areas, unbuilt spaces among houses, curbs of streets and even on large avenues. These owls are in constant contact with garden and house owners, children, pedestrians and intense car traffic. On the other hand, predation pressure is much higher in rural than in urban owls (authors, upubl. data). There is not a clear habitat interface between urban and rural habitats, since urbanized areas are immediately surrounded by large, private fenced areas of grasslands, pastures and cereal crops that are not inhabited by humans. Moreover, despite that some rural territories are in close proximity to urban ones (Fig. 4), breeding dispersal rates and dispersal distances are extremely low and none of the banded adults dispersed between urban and rural habitats in this study (Authors unpub. data).

**Figure 5:**
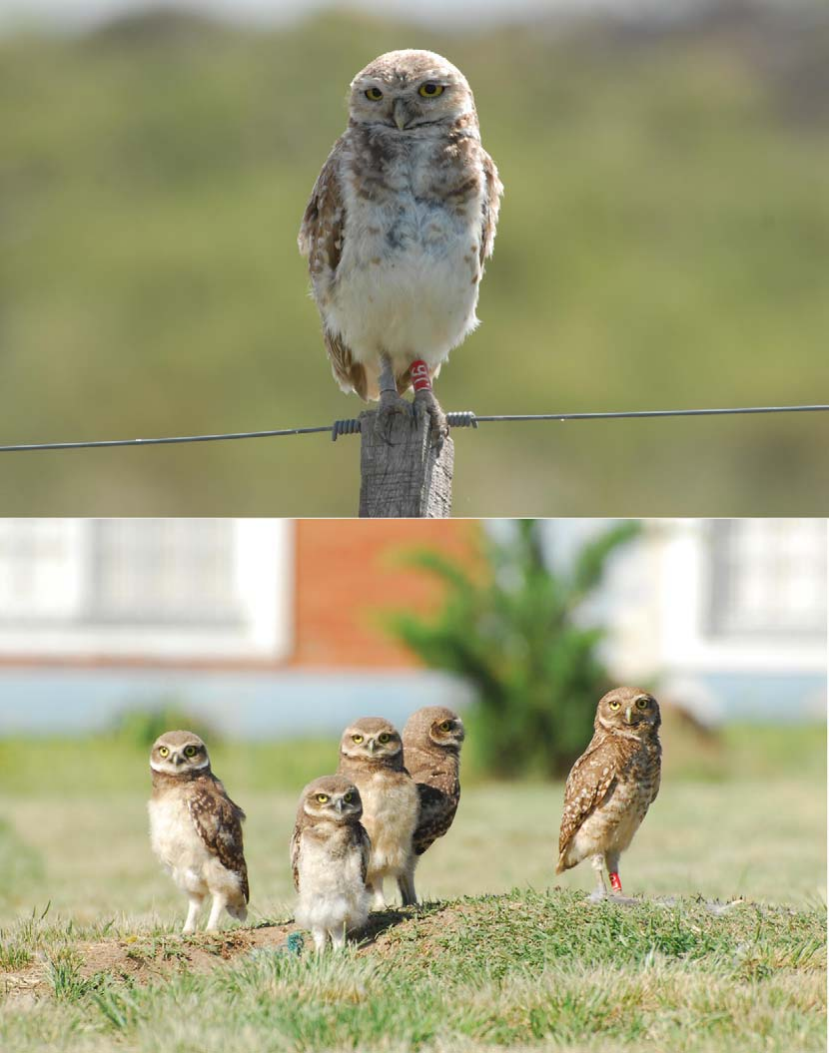
Top: adult male burrowing owl banded in a rural territory. Bottom: adult banded female attending four fledglings in an urban nest. Plumage differences among sexes and ages allow identification. Photography taken by J.L. Tella.

We trapped >1,000 adults and chicks since 2006 using bow nets and ribbon carpets to mark them with a plastic colour-numbered ring readable at a distance. Pair members were sexed based on plumage patterns and colouration^19^ (Fig. 5), and sex was later confirmed by molecular analyses (Authors, unpubl. data). FIDs were measured by the same person (JLT) in a sample of 338 banded owls when they were rearing chicks (from late November to late January) during five consecutive breeding seasons (2008–2012). We chose the breeding season, not considering potential seasonal changes in FID, since it is during this period when fear of humans could preclude some birds from reproducing in human-disturbed habitats^17^. The high territory fidelity across seasons and years and low breeding dispersal of the species in the study area (authors, unpubl. data), together with a high monitoring effort of territories, allowed high inter-year resighting probabilities of banded owls and thus the monitoring of FIDs during consecutive years over their lifespan. The standard procedure used was to walk towards focal individuals, which were perched close to their nests, following a direct trajectory, with no obstacles blocking the bird and the observer and at a constant speed of 0.5 m/s. Distances at which birds fled were measured using a laser telemeter incorporated into 10 × 42 binoculars (Leica Geovid, range: 10–1300 m) or counting paces for distances of less than 10 m^19^. FIDs were measured during the day, when owls were easily located at a distance, given the bare ground and short vegetation surrounding their nests. We performed successive trials (2–3 per individual separated by at least 3 days) within each breeding season to calculate an average value per individual and year. However, as burrowing owls show high intra-year repeatability in this behaviour (r = 0.84–0.92)^19^, we also considered individuals for which we could only record one FID. This information is important in order to capture inter-individual variability^24^, but as our sample size is large enough results remained similar when data sets were built including or excluding birds with a single FID (results not shown). Although FID is considered as an anti-predator behaviour, we found that individuals responded differently when exposed to a terrestrial predator, an aerial predator, and an approaching human (Authors unpublish. data). Thus, FID measured using the described protocol could be considered as a specific descriptor of fear of humans.

### Statistical analysis

We investigated intra-individual consistency in FID (log-transformed) as well as factors influencing its inter-individual variability using Generalized Linear Mixed Models (normal error distribution, identity link function). Whenever FIDs of some individuals were measured in different years and these inter-annual trials were measured in different contexts (territories), we first examined the optimal random effect structure testing whether or not individuals were nested within territories^42^. The inclusion of territory in the random structure of models also allows us to control for potential environmental differences among breeding sites, which could not be accurately measured^42^. Models performed considering the clustering distribution of owls (individuals nested within territories and clusters) were indistinguishable (in terms of changes in AIC) from our best-supported models (results not shown).

We started with a model where the fixed component contained all explanatory variables (the *beyond optimal model*) to find the optimal structure of the random component using Restricted Maximum Likelihood (REML) estimators and comparing AIC_REML_ values. Once the optimal random structure was found, we modelled the fixed term and compared models using Maximum Likelihood (ML) estimators. Final models and repeatabilities are presented using REML estimators^42^. Then, we considered whether or not FID consistently varied between individuals in successive years by comparing a random intercept and a random intercept plus slope model. The first model assumes that all individuals, even when they vary in their average level of fear (intercept), behave similarly when they are repeatedly measured (i.e., all of them increase, decrease or do not change their fear of humans). The second approach (also called reaction norm approach) estimates individual variation in an average behaviour (individual variation or “V_i_”) while taking into account differences in behavioural plasticity (individual × environment interaction or “I × E”). For each individual, average annual FIDs were sequentially numbered, and the test number was used as the environmental variable, which reflects the accumulated exposure of individuals to humans with years. In the case of known-age birds tested from their first-year onwards (see below), the test number equals their age in years. Evidence for the presence of a reaction norm (I × E) was assessed by comparing through a likelihood-ratio test^42^ a random intercept and slope model against a model that only included variance in elevation (random intercept model). All random intercepts and slopes were modelled as normally-distributed random variables with zero mean and variance (σ^2^). We calculated the power of our data structure to detect V_i_ and IxE, as well as the sample size necessary to properly test them using simulations^24^. Sex and habitat (rural versus urban) were fitted as fixed factors given gender and habitat differences in FIDs of burrowing owls^19^. Moreover, we used a sub-sample of individuals banded as chicks to test the extent to which birds changed their FID since their first reproductive attempt (the rest of the tested individuals were banded as adults of unknown age). Models were selected using Akaike Information Criteria (AIC). Smaller AICc values suggest a better fit of the model to the data, and models whose AIC values differ from that of the top model (ΔAIC) by more than two points are considered to lack explanatory power relative to the top model^42^.

Repeatability of FID was estimated as r = σ^2^_α_ / (σ^2^_α_ + σ^2^_ϵ_), where σ^2^_α_ and σ^2^_ϵ_ can be extracted directly from the output of mixed models^43^. The standard errors of repeatability values were estimated following Becker^44^. To test the null-hypothesis that r = 0, we randomized the response vector (FID) a large number of times (n = 1,000). Randomizations were conducted considering differences in FID between rural and urban birds as well as among sexes (see results). The appropriate *p* value was the proportion of randomizations that yielded *r*_randomized_ ≥ *r*_observed_. 95% CI obtained in simulations are provided.

## Figures and Tables

**Table 1:** GLMMs built to explain variability in FID of burrowing owls. Repeatability (r ± SE) is shown for each model as well as 95% confidence intervals obtained through simulations. Models are ranked by AIC, obtained through restricted maximum likelihood (REML; comparison of models with identical fixed covariates but different random structures) or maximum likelihood (ML; comparison of models with identical random structures but different fixed covariates). Models with ΔAIC > 15 were not included

All birds									
Random effects	Fixed effects	AIC_ML_	ΔAIC_ML_	AICw_ML_	AIC_REML_	ΔAIC_REML_	AICw_REML_	r (±SE)	95% CI of simulations
Individual(territory)	sex, habitat, years	−229.59	0	0.504	−211.66	0	0.79	0.91 (0.0001)	0–0.12
	years, habitat	−229.04	0.55	0.383					
	Habitat	−226.6	2.99	0.113					
Individual	sex, habitat, years	−226.36	0	0.651	−208.98	2.68	0.21	0.85 (0.0003)	0–0.11
	years, habitat	−225.11	1.25	0.348					

Urban birds									
Random effects	Fixed effects	AIC_ML_	ΔAIC_ML_	AICw_ML_	AIC_REML_	ΔAIC_REML_	AICw_REML_	r (±SE)	95% CI of simulations
Individual(territory)	Years	−195.52	0	0.469					
	sex, years	−194.68	0.84	0.308	−180.92	0	1	0.90 (±0.0002)	0–0.12
	Sex	−194.04	1.48	0.224					
Individual	sex, years				−170.61	10.31	0.01	0.83 (±0.0005)	0–0.11

Rural birds									
Random effects	Fixed effects	AIC_ML_	ΔAIC_ML_	AICw_ML_	AIC_REML_	ΔAIC_REML_	AICw_REML_	r (±SE)	95% CI of simulations
Individual	Years	−54.45	0	0.603					
	sex, years	−53.52	0.93	0.379	−42.83	0	1	0.96 (±0.0001)	0–0.34
	Sex	−47.49	6.96	0.019					
Individual(territory)	sex, years				−28.17	14.66	0	0.97 (±0.0001)	0–0.35
